# Global phylogenomics of multidrug-resistant *Salmonella enterica* serotype Kentucky ST198

**DOI:** 10.1099/mgen.0.000269

**Published:** 2019-05-20

**Authors:** Jane Hawkey, Simon Le Hello, Benoît Doublet, Sophie A. Granier, Rene S. Hendriksen, W. Florian Fricke, Pieter-Jan Ceyssens, Camille Gomart, Helen Billman-Jacobe, Kathryn E. Holt, François-Xavier Weill

**Affiliations:** 1 Department of Biochemistry and Molecular Biology, Bio21 Molecular Science and Biotechnology Institute, University of Melbourne, Parkville, Victoria 3010, Australia; 2 Department of Infectious Diseases, Central Clinical School, Monash University, Melbourne, Victoria 3004, Australia; 3 Unité des Bactéries Pathogènes Entériques, Centre National de Référence des * Escherichia coli*, * Shigella * et * Salmonella *, World Health Organization Collaborative Centre for the Typing and Antibiotic Resistance of * Salmonella *, Institut Pasteur, 75015 Paris, France; 4 ISP, Institut National de la Recherche Agronomique, Université François Rabelais de Tours, UMR 1282, Nouzilly, France; 5 Laboratoire de sécurité des aliments, Agence Nationale de Sécurité Sanitaire de l'Alimentation, de l'Environnement et du Travail (ANSES), Université PARIS-EST, 94701 Maisons-Alfort, France; 6 Laboratoire de Fougères, Agence Nationale de Sécurité Sanitaire de l'Alimentation, de l'Environnement et du Travail (ANSES), 35306 Fougères, France; 7 Research Group for Genomic Epidemiology, National Food Institute, Technical University of Denmark, Kongens Lyngby, Denmark; 8 Department of Microbiome Research and Applied Bioinformatics, University of Hohenheim, Stuttgart, Germany; 9 Institute for Genome Sciences, University of Maryland School of Medicine, Baltimore, MD, USA; 10 Bacterial Diseases Unit, Sciensano, Brussels, Belgium; 11 Asia-Pacific Centre for Animal Health, Faculty of Veterinary and Agricultural Science, University of Melbourne, Parkville, Victoria 3010, Australia; 12 London School of Hygiene and Tropical Medicine, London WC1E 7HT, UK

**Keywords:** *Salmonella*, phylogenomics, MDR, Kentucky, SGI, ST198

## Abstract

*
Salmonella enterica* serotype Kentucky can be a common causative agent of salmonellosis, usually associated with consumption of contaminated poultry. Antimicrobial resistance (AMR) to multiple drugs, including ciprofloxacin, is an emerging problem within this serotype. We used whole-genome sequencing (WGS) to investigate the phylogenetic structure and AMR content of 121 *S.* *e* *nterica* serotype Kentucky sequence type 198 isolates from five continents. Population structure was inferred using phylogenomic analysis and whole genomes were compared to investigate changes in gene content, with a focus on acquired AMR genes. Our analysis showed that multidrug-resistant (MDR) *S.* *enterica* serotype Kentucky isolates belonged to a single lineage, which we estimate emerged circa 1989 following the acquisition of the AMR-associated *Salmonella* genomic island (SGI) 1 (variant SGI1-K) conferring resistance to ampicillin, streptomycin, gentamicin, sulfamethoxazole and tetracycline. Phylogeographical analysis indicates this clone emerged in Egypt before disseminating into Northern, Southern and Western Africa, then to the Middle East, Asia and the European Union. The MDR clone has since accumulated various substitution mutations in the quinolone-resistance-determining regions (QRDRs) of DNA gyrase (*gyrA*) and DNA topoisomerase IV (*parC*), such that most strains carry three QRDR mutations which together confer resistance to ciprofloxacin. The majority of AMR genes in the *S*. *e* *nterica* serotype Kentucky genomes were carried either on plasmids or SGI structures. Remarkably, each genome of the MDR clone carried a different SGI1-K derivative structure; this variation could be attributed to IS*26*-mediated insertions and deletions, which appear to have hampered previous attempts to trace the clone’s evolution using sub-WGS resolution approaches. Several different AMR plasmids were also identified, encoding resistance to chloramphenicol, third-generation cephalosporins, carbapenems and/or azithromycin. These results indicate that most MDR *S*. *e* *nterica* serotype Kentucky circulating globally result from the clonal expansion of a single lineage that acquired chromosomal AMR genes 30 years ago, and has continued to diversify and accumulate additional resistances to last-line oral antimicrobials. This article contains data hosted by Microreact.

## Data Summary

All sequencing reads generated in this study have been deposited in the European Nucleotide Archive (ENA) (http://www.ebi.ac.uk/ena) under project number PRJNA445436. SRA accession numbers can be found in Table S1 (available in the online version of this article). The reference genome sequence for *Salmonella* *enterica* serotype Kentucky strain 201001922 has been deposited in GenBank under accession number CP028357. The phylogeny and associated metadata can be viewed on Microreact: https://microreact.org/project/Hkl7CzEXV.

**Impact Statement**

Fluoroquinolone-resistant *Salmonella enterica* and carbapenem-resistant, extended-spectrum *β*-lactamase producing *Enterobacteriaceae* are amongst the highest priority pathogens posing a risk to human health as determined by the World Health Organization (WHO). All of these high level resistances have been detected in a single serotype of *S. enterica *, serotype Kentucky, against a background of multidrug resistance to first-line antimicrobials, leaving very limited treatment options. Here, we analysed the genomes of *S*. *e* *nterica* serotype Kentucky from geographically diverse sources, to investigate the emergence and spread of antibiotic resistance in this problem pathogen. We discovered that the multidrug-resistant (MDR) genomes in our collection comprised a clonal MDR lineage that we estimate arose in Egypt in ~1989, before spreading across Africa, then into Europe, the Middle East and Asia. Resistance to first-line antibiotics mostly arose from the chromosomal integration of a large genomic island, the *Salmonella* genomic island 1, in the common ancestor of the MDR lineage. Most strains were also fluoroquinolone resistant, due to acquisition of point mutations in chromosomal genes *gyrA* and *parC* early in the clone’s evolution. Additional resistances, including to third-generation cephalosporins (such as ceftriaxone), carbapenems (such as imipenem) and the last-line oral antibiotic azithromycin, emerged through acquisition of diverse locally circulating MDR plasmids. Aside from antibiotic resistance, we found no other genetic determinants that could explain the global success of this *S*. *e* *nterica* serotype Kentucky lineage. These data show the MDR clone of *S*. *e* *nterica* serotype Kentucky is already widespread and is capable of acquiring last-line resistances, suggesting it should be considered a high-risk global MDR clone.

## Introduction

Carbapenem-resistant, extended-spectrum *β*-lactamase (ESBL) producing *Enterobacteriaceae* and fluoroquinolone-resistant *Salmonella* have been recently listed as priority pathogens that pose the greatest threats to human health (critical and high threat levels, respectively) by the World Health Organization (WHO) [1]. All these resistances have been observed in a single serotype of *Salmonella enterica *, serotype Kentucky, since the 2000s [2–5]. Ciprofloxacin-resistant (CIP^R^) *S*. *enterica* serotype Kentucky was first observed in a French traveller returning from Egypt in 2002, before being increasingly isolated globally [2]. Between 2007 and 2012, the European Centers for Disease Control and Prevention (ECDC) reported 1301 isolations of *S*. *e* *nterica* serotype Kentucky from 12 countries, including 955 (73.4 %) CIP^R^ *S*. *e* *nterica* serotype Kentucky [6]. These isolates were found in patients across the world, but predominantly in Northern Africa, Europe and Southern Asia. Several previous studies have described the rapid spread of CIP^R^ *S*. *enterica* serotype Kentucky from Northern Africa to the rest of the African continent, as well as the Middle East, Europe and Asia [3–5]. CIP^R^ *S*. *e* *nterica* serotype Kentucky is a foodborne pathogen that causes gastroenteritis in humans, and domestic poultry has played an important role in its global spread (most recently in South Asia and Europe). Multilocus sequence typing (MLST) and PFGE have revealed that CIP^R^ *S*. *e* *nterica* serotype Kentucky is a single population belonging to sequence type (ST)198 and not ST152, which is a prevalent *S*. *e* *nterica* serotype Kentucky ST found in poultry in the USA but rarely reported in humans [7].

Before the 1990s, *S*. *enterica* serotype Kentucky ST198 was susceptible to all antibiotics. Since then, multidrug resistance has emerged [2]. In the early 1990s, *S*. *e* *nterica* serotype Kentucky ST198 acquired a variant of the *Salmonella* genomic island (SGI) 1 in the chromosome, likely in Egypt [8]. Initially characterized in *S. enterica* serotype Typhimurium strain DT104 [9], the SGI1 is a site-specific integrative mobilizable element (IME) that integrates in the 3′-end of the conserved chromosomal gene *trmE* [10]. SGI1 is the prototype element of a multidrug resistance IME family named SGI/PGI/AGI, which includes both *Proteus* genomic islands (PGIs) [11] and *Acinetobacter* genomic islands (AGIs) [12]. They consist of a 27 kbp related backbone with conserved gene synteny and variable regions containing complex class 1 integron structures, insertion sequences and transposon elements that are responsible for multidrug resistance. As an IME, SGI1 is specifically mobilized *in trans* by conjugative IncC plasmids [13–15]. The most recent findings revealed complex interactions between SGI1 and IncC plasmids for transfer and maintenance. Since the first description of SGI1 in *S*. *enterica* serotype Typhimurium DT104, several variants of SGI/PGI/AGI have been discovered, which differ in their antimicrobial resistance (AMR) gene content and AMR gene cluster structure [16, 17] in species of families *Enterobacteriaceae* and *Morganellaceae *, and *Acinetobacter baumannii* [12, 18]. These variants usually differ in the composition of the integron, and each variant carries different AMR genes. One variant of the SGI, known as SGI2 or SGI1-J, differs not only in the composition of the integron, but also in the site at which the integron is inserted into the SGI backbone [8, 19].

Four main types of SGI have so far been described in *S*. *enterica* serotype Kentucky: SGI1-K, SGI1-P, SGI1-Q and SGI2 [4]. These SGI1 variants share a common genetic feature consisting of an insertion/deletion between *S005* and *S009* due to the insertion of IS*1359*, which was also found in a few other SGI1 variants in strains of different *S. enterica* serotypes isolated in 2000 in Egypt, and more recently in *Proteus mirabilis* [20]. Additionally, these three SGI1 variants show a truncation at the 5′-end of S044, the final ORF of the SGI backbone, through the insertion of IS*26* [21]. SGI1-K contains a complex mosaic resistance region made of different segments of transposons Tn*21*, Tn*1721*, Tn*5393*, Tn*3*-like and a In4-type integron structure, as well as IS*26* elements [22]. SGI1-P and SGI1-Q contain only the IS*26*-flanked Tn*3*-like structure carrying *bla*_TEM-1_ and only the rightmost IS*26* in S044, respectively [21].

After the acquisition of SGI1 by the multidrug-resistant (MDR) lineage, high level-resistance to fluoroquinolones emerged, conferred by a combination of three amino-acid substitutions in the quinolone-resistance-determining region (QRDR) of *gyrA* and *parC*. Previous epidemiological studies determined that these mutations likely arose in Egypt in the early 2000s [3].

Finally, additional resistance was gained through the acquisition of locally circulating plasmid-borne ESBL, AmpC and/or carbapenemase genes [4]. Additionally, the geographical distribution of CIP^R^ *S*. *enterica* serotype Kentucky ST198 overlaps with other highly drug resistant *Enterobacteriaceae* carrying plasmid-borne ESBL, AmpC and/or carbapenemase genes, leading to predictions that highly-drug resistant *S*. *e* *nterica* serotype Kentucky ST198 strains are likely to become more frequent in the near future due to novel plasmid acquisitions [4, 5].

To date, all previous studies have used conventional typing methods [MLST, PCR, PFGE and antimicrobial susceptibility testing (AST)] and together they suggest that the recent global spread of CIP^R^ *S*. *enterica* serotype Kentucky may reflect the expansion of a single clone, driven by the emergence of AMR. However, the precise nature, order and timing of the evolutionary events underlying this overall picture, remain unclear. Here, we investigated the global population structure of MDR *S*. *enterica* serotype Kentucky ST198 using whole-genome sequencing (WGS) and phylogenomic analysis to interrogate a collection of 121 human and non-human isolates collected from 33 countries on five continents, between 1937 and 2016. We used comparative genomics to reconstruct the various steps in the acquisition of AMR determinants within the emerging MDR *S*. *e* *nterica* serotype Kentucky ST198 clone, and to investigate the presence of genetic elements not related to AMR that might have conferred other selective advantages to this emerging bacterial pathogen.

## Methods

### Bacterial isolates used in this study

A total of 97 *S*. *enterica* serotype Kentucky ST198 isolates were directly analysed in this study (Table S1), including 68 isolates collected between 1937 and 2013 that were previously studied by conventional molecular methods [3–5, 23], and 29 new isolates collected between 2008 and 2016. These isolates originated from the French National Reference Center for *Escherichia coli *, *Shigella *, *Salmonella* (Institut Pasteur) and several other international laboratories, and were selected on the basis of their diversity (human or non-human source, geographical area and year of isolation, PFGE types, and AMR phenotypes and genotypes). WGS data for a further 24 *S*. *enterica* serotype Kentucky isolates was included in genomic analyses as detailed below.

### AST

AST was performed on all 97 *S*. *enterica* serotype Kentucky ST198 isolates using the disc diffusion method with a panel of 32 antimicrobial agents (Bio-Rad), as described previously [24]. The minimum inhibitory concentrations (MICs) of ceftriaxone, ceftazidime, imipenem, ertapenem, meropenem, ciprofloxacin, azithromycin and tigecycline were also determined by Etests (AB Biodisk). Results were interpreted with the Antibiogram Committee of the French Society for Microbiology/European Committee on Antimicrobial Susceptibility Testing (CA-SFM/EUCAST) (www.sfm-microbiologie.org/) breakpoints. In particular, we used ciprofloxacin clinical breakpoints defined for intestinal *Salmonella* isolates: susceptible when MIC≤0.25 mg l^−1^, and resistant when MIC>0.5 mg l^−1^.

### WGS

The 97 *S*. *enterica* serotype Kentucky ST198 isolates were subjected to WGS with Illumina at GATC Biotech, Germany (Illumina HiSeq) (*n*=45), the Institut Pasteur, France (PF1 and P2M sequencing platforms, Illumina HiSeq and NextSeq, respectively) (*n*=43), the Technical University of Denmark, Denmark (*n*=7, Illumina MiSeq) or at the Institute for Genome Sciences, University of Maryland School of Medicine (IGS-UoM), USA (Illumina HiSeq) (*n*=2). Paired-end reads varied in read length depending on the sequencing platform/site, from 100 to 146 bp, yielding a mean of 196-fold coverage per isolate (minimum 30-fold, maximum 687-fold) (Table S1). Short-read sequences have been deposited at the European Nucleotide Archive (ENA) (http://www.ebi.ac.uk/ena), under study accession number PRJNA445436 and the genome accession numbers are provided in Table S1.

### Other genomes studied

Additional *S*. *enterica* serotype Kentucky ST198 WGS data were obtained from the GenomeTrakr project (https://ftp-trace.ncbi.nih.gov/pathogen/Results/Salmonella) [25, 26]. All 3014 *S*. *e* *nterica* serotype Kentucky isolates in the *Salmonella* project were downloaded from National Center for Biotechnology Information on 06/01/2016, and ST was determined using srst2 [27]. From the 73 available ST198 GenomeTrakr sequences, we excluded those that were missing the source information required for our analysis (source, location and year of isolation), and retained those from geographical regions underrepresented in our own dataset that were non-redundant in terms of source/outbreak (*n*=24; accession numbers in Table S1), bringing the total number of genomes analysed in this study to 121.

### Sequencing and construction of reference genome 201001922

Genomic DNA from *S*. *enterica* serotype Kentucky ST198 isolate 201001922 was also sequenced using a hybrid sequencing approach at the Institute for Genome Sciences, University of Maryland School of Medicine (IGS-UoM), USA, as described elsewhere [28]. Paired-end, 3 kb insert libraries sequenced on the 454 GS FLX Titanium platform (Roche) were combined with paired-end, 300 to 400 bp insert libraries sequenced with 100 bp read length on the HiSeq 2000 platform (Illumina). Hybrid assemblies were generated with the Celera assembler (http://wgs-assembler.sourceforge.net/wiki/) based on different ratios of 454 and Illumina sequence data, and the outputs were compared with respect to the number of resulting scaffolds and total scaffold length. For the final assembly, a 27-fold genome coverage of 454 data and a 30-fold coverage of Illumina sequence data were combined to create a draft genome sequence consisting of 11 scaffolds and a total length of 4.86 Mbp.

Contigs and scaffolds from the draft assembly were concatenated using a linker sequence (*NNNNNCACACACTTAATTAATTAAGTGTGTGNNNNN*), in order to generate continuous ‘pseudochromosomes’. The linker sequence contains START and STOP codons in each frame and orientation, to allow the gene finder to call truncated genes at all contig ends. Contig orders and orientations within the pseudochromosome were determined based on NUCmer v3.23 [29] nucleotide sequence comparison to ST152 *S*. *enterica* serotype Kentucky strain CVM29188 (SL475) as a reference genome. Protein-coding and RNA gene predictions and functional annotations were carried out with CloVR-Microbe [30]. The genome sequence of *S*. *e* *nterica* serotype Kentucky ST198 isolate 201001922 has been deposited in GenBank under the accession number CP028357.

### Mapping and phylogenomic analysis

Short reads for all 121 *S*. *e* *nterica* serotype Kentucky ST198 isolates were mapped to the reference genome 201001922 using the mapping pipeline RedDog v1b4 (https://github.com/katholt/RedDog) to identify single-nucleotide variants (SNVs), as previously described [31, 32]. RedDog uses Bowtie2 v2.2.3 [33] with the sensitive local method and a maximum insert size of 2000 to map all genomes to the reference genome. SNVs were then identified using SAMtools v0.0.19 [34] with a Phred score ≥30, and alleles at each locus were determined by comparing to the consensus base in that genome, using SAMtools pileup to remove low quality alleles (Phred base quality ≤20, read depth ≤5 or a heterozygous base call). SNVs were filtered to exclude those present in repeat regions, phage regions or the SGI. Gubbins v1 [35] was run using default settings to identify and remove SNVs in recombinant regions. The final SNV set used for phylogenetic analysis consisted of 2066 SNVs.

To estimate a Bayesian phylogeny with divergence dates, an alignment of SNV alleles was passed to beast (Bayesian Evolutionary Analysis Sampling Trees) v2.4.6 [36], in addition to isolation dates for each genome. The model parameters were as follows: GTR+G substitution model, lognormal relaxed clock, constant population size. As the coefficient of rate variation parameter was calculated to be 0.57 (95 % highest posterior density (HPD) 0.44–0.70), and the distribution was not abutting zero, a relaxed clock model was favoured over a strict clock. The model with a constant population size produced higher overall likelihoods compared to a Bayesian skyline model, and calculations of changes in population size in the skyline model indicated that the population had been constant over time, so the simpler model was favoured. Five independent beast runs of 100 million iterations were combined, representing 450 million Markov chain Monte Carlo (MCMC) generations after burn-in removed. Parameter estimates were calculated using Tracer v1.6 [37]. A maximum clade credibility tree was generated using TreeAnnotator v1.7.5 [38]. To test the robustness of the molecular clock signal, ten further beast runs with randomized tip dates were generated using the same model.

Additional testing of the molecular clock was undertaken by constructing a maximum-likelihood phylogeny using RAxML v8.1.23 [39], using 100 bootstrap replicates, with the final set of SNVs. To check for a molecular clock signal, a linear regression was performed using the root-to-tip distances from the phylogeny with year of isolation. Phylogeographical analysis was performed by modelling geographical region (defined by the United Nations subregion geoschemes [40]) as a discrete trait on the final beast tree, using an empirical Bayes method [41] implemented in the make.simmap function in *phytools* v0.6.44 [42].

### Assembly, annotation and pangenome analysis

All reads were filtered using FastXToolKit v0.0.14 [43] to remove all reads containing bases called as ‘N’, and Trimmomatic v0.30 [44] was used to remove any reads with a mean Phred quality score below 30. Each isolate genome was assembled using SPAdes v3.5 [45] using a kmer range of 21, 33, 55, 65 and 75. Scaffolding was performed using SSPACE v3.0 [46] and GapFiller v1.10 [47] with default settings. All assemblies were ordered against the *S*. *e* *nterica* serotype Kentucky ST198 strain 201001922 reference genome using Abacas v1.3.1 [48]. Prokka v1.10 [49] was used to annotate each assembly using a preferential protein database made up of coding sequences from the 201001922 reference genome, the ARG-Annot resistance database [50], and the SGI1, SGI1-K and SGI2 references (accession numbers AF261825, AY463797 and AY963803). Roary v3.6.0 [51] was used to determine core and accessory genes for all annotated genomes. Core genes were defined as present in at least 95 % of genomes.

### Identification of resistance, virulence and phage genes

AMR gene alleles were determined by mapping short reads to the ARG-Annot resistance database [50] using srst2 [27]. AMR gene locations were determined by interrogating genome assemblies with blast v2.3.0 [52]. Associations between AMR genes and SGI type or geographical regions were determined using two-way contingency tables for each gene. Each region was tested with Fisher’s exact test to determine whether the frequency of the gene was positively associated with that specific region compared to all other regions. A *P* value cut-off of 0.05 was used to determine significance.

Presence or absence of *Salmonella* virulence genes defined in the vfdb database [53] was determined using srst2 to screen the short-read data. All genomes were screened using phaster [54] to detect phage regions.

### Reconstruction of SGI sequences

ISMapper v1 [55] and the assembly graph viewer Bandage [56] were used to piece together segments of the SGI in each genome. To do this, each assembly was queried with blast to identify which contigs contained SGI backbone and AMR genes. Each assembly was also queried for IS*26* using ISMapper's assembly improvement mode [55], identifying contigs that contained IS*26* flanking sequence. Contigs containing flanking IS*26* sequence with SGI genes or AMR genes were hypothesized to be part of the SGI. Both pieces of information (blast and ISMapper results) were used in conjunction with the reference SGI1-K reference sequence (accession number AY463797) to determine which contigs could be joined together. In some cases, it was unclear whether IS*26*-flanked AMR genes were located within the SGI or a plasmid. In these cases, Bandage was used to examine the assembly graphs and determine the paths linking the SGI, IS*26* and AMR genes, providing additional evidence for contig connection.

IS*26* copy number was estimated by mapping all genomes to the IS*26* sequence using Bowtie v2.2.9 [33], and dividing the read depth across IS*26* by the mean chromosomal read depth. To assess whether IS*26* copy number was increasing over time within the MDR lineage, a linear regression analysis was performed using estimated IS*26* copy number and year of isolation for each isolate.

### Analysis of IncI1 and IncC plasmids

All *S*. *e* *nterica* serotype Kentucky ST198 genomes were screened for plasmid replicons using srst2 v0.2.0 with the version of the PlasmidFinder database [57] that is distributed in the srst2 package. Reads from *S*. *e* *nterica* serotype Kentucky ST198 isolates containing IncI1 plasmids as well as a set of publicly available IncI1 plasmid sequences (Table S2) were mapped to the IncI1 plasmid pNF1358 (accession number DQ017661). SNVs were called using the same method as described above for chromosomal SNVs. The resulting SNVs were filtered to include only those that were present in core genes (defined as genes present in 100 % of the IncI1 plasmid sequences, see Table S3). The final alignment consisted of 1380 SNVs, which was used to create a maximum-likelihood tree with RAxML v8.1.16 [39] using a GTR+G model with 100 bootstraps. Reads from *S*. *e* *nterica* serotype Kentucky ST198 isolates containing IncC plasmids were typed with srst2 against the cgMLST IncA/C plasmid database [58] to determine the 28-locus plasmid sequence type (pST) for each plasmid.

## Results

### Phylogenetic analysis of *S*. *e* *nterica* serotype Kentucky ST198

**Fig. 1.**
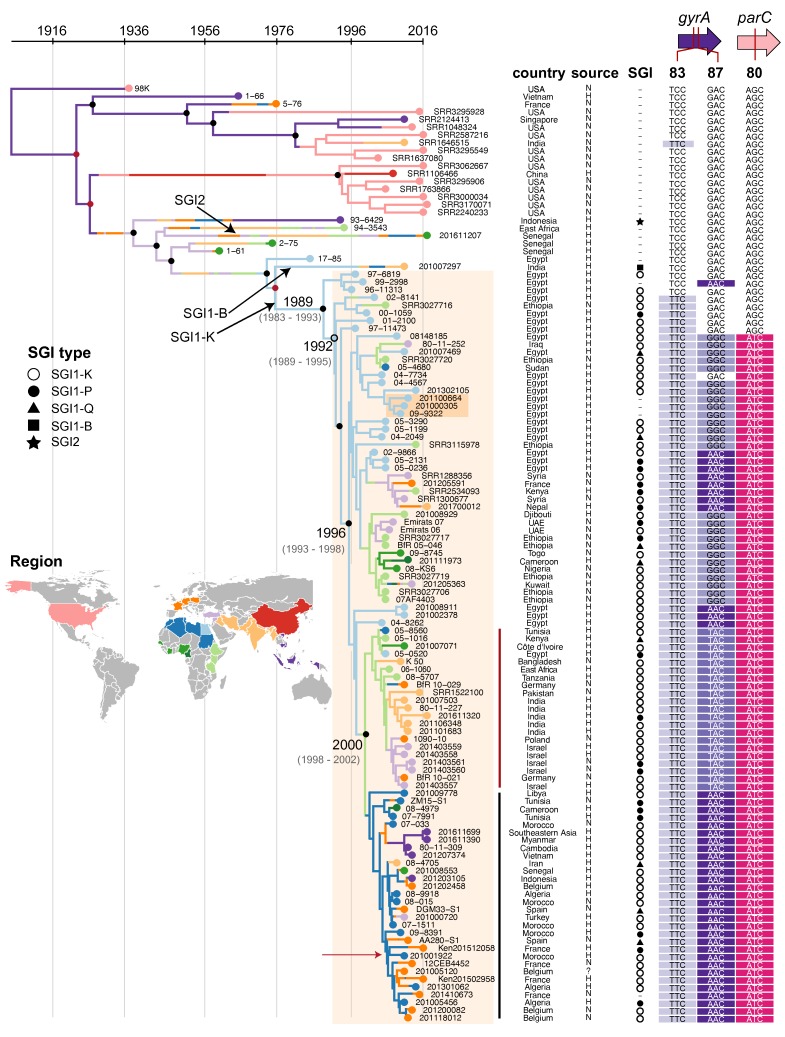
Phylogeographical analysis of *S.* *e* *nterica* serotype Kentucky ST198 based on whole-genome SNV data. Bayesian maximum clade credibility tree inferred using beast, with the MDR lineage shaded orange. The dark orange box indicates three isolates from the same patient. Major internal nodes are labelled with circles indicating branch support (black, ≥95 % posterior support; red, >70 % posterior support; unfilled, >30 % posterior support); divergence date estimates (95 % higher posterior density values) are provided for key points in the evolution of the MDR lineage. Leaf nodes are coloured by region of origin (see inset map). Coloured branches indicate inferred geographical distribution of internal branches, inferred using maximum-likelihood ancestral trait reconstruction. Data columns indicate country of origin; source of isolate (H for human, N for non-human, ? for unknown); SGI type (see inset legend); quinolone resistance-related codons, with resistance-associated alleles highlighted. Reference genome 201001922 is marked with red arrow. Red and black vertical lines indicate clades that are mentioned in the text.

All 121 *S*. *e* *nterica* serotype Kentucky ST198 genomes were mapped to the draft reference genome for *S*. *e* *nterica* serotype Kentucky ST198 strain 201001922 (see Methods), and 2066 SNVs were identified in the core genome. Linear regression of root-to-tip distances against year of isolation indicated strong temporal structure for all isolates, as did date randomization tests in beast (Figs S1 and S2). The alignment of these SNVs and the years of isolation were then used to construct a dated phylogenetic tree using beast, which was further overlaid with region of origin to infer routes of geographical spread (see Methods). The results (Fig. 1) indicate that nearly all MDR isolates belong to a single monophyletic clade of *S*. *e* *nterica* serotype Kentucky ST198, which we estimate emerged around 1989 (95 % HPD 1983–1993) in Egypt (Fig. 1). The beast analysis estimated the evolutionary rate to be 4.8×10^−7^ substitutions per site per year (95 % HPD 5.28×10^−7^−3.78×10^−7^ substitutions per site per year; see Fig. S2). This is equivalent to a mean rate of 1.6 SNVs per year, which is similar to rates estimated for other nontyphoidal *Salmonella* serotypes including Typhimurium and Agona [59–61], and faster than those estimated for typhoidal serotypes Typhi and Paratyphi A [62–64].

The MDR clade includes all isolates carrying SGI1-K and derived variants, which include all of the CIP^R^ *S*. *e* *nterica* serotype Kentucky ST198 (Fig. 1; more details below). In addition to the SGI, the MDR lineage has accumulated amino acid mutations in the QRDR. The first mutation occurred circa 1992 in *gyrA* codon 83 (TCC to TTC, Ser83Phe) (light purple, Fig. 1), and was then followed circa 1996 by a mutation in codon 80 of *parC* (AGC to ATC, Ser80Ile) (pink, Fig. 1). These mutations increased MIC for ciprofloxacin, but CIP^R^ did not arise until additional mutations in codon 87 of *gyrA* occurred; at least three such mutations were observed in the MDR clade (GAC to GGC, AAC or TAC; Asp87Gly, Asp87Asn, Asp87Tyr) (dark purple shading, Fig. 1).

The *parC*-80 and *gyrA*-87 mutations accompanied a dramatic clonal expansion, with the clone spreading from Egypt to other geographical locations (Fig. 1). Multiple independent transfers of *S*. *e* *nterica* serotype Kentucky ST198 out of Egypt and Northern Africa are evident, with two clades, carrying either Asp87Tyr (TAC) or Asp87Asn (AAC) mutations in GyrA codon 87, emerging circa 2000. The former spread into East Africa, Middle Africa, South Asia, Europe and Western Asia (dark red line, Fig. 1); the latter spread to South-East Asia, Europe and West Africa (black line, Fig. 1).

Interestingly, the ST198 genomes isolated from agricultural sources in the USA (including 98K, isolated from poultry in 1937, see Table S1) lack the SGI and *gyrA/parC* mutations (Fig. 1). Notably, while these strains were isolated contemporaneously with the MDR clade (2003 to 2016) they are only distantly related to it, sharing a most recent common ancestor (MRCA) circa 1925 (95 % HPD 1898–1938; Fig. 1). This finding is consistent with previous work indicating that ST198 isolates from livestock or poultry in the USA belong to a different genomic cluster (198.1) than MDR ST198 isolates from clinical cases (198.2) [7].

### Long-term persistence in a single patient

Three *S*. *e* *nterica* serotype Kentucky ST198 isolates were recovered in consecutive years (2009, 2010 and 2011) from the same patient, who had been infected in Egypt (dark orange box, Fig. 1). These isolates belonged to the MDR lineage and shared an MRCA circa 2005, suggesting persistent colonization of ~6 years duration (Fig. 1). The 2011 isolate, 201100664, differed the most from the inferred MRCA (30 SNVs; 21 non-synonymous SNVs, 6 synonymous SNVs, 3 intergenic SNVs), yielding an estimated *in vivo* substitution rate of 5 SNVs per year, faster than that estimated by beast analysis of the whole data set. Many of the non-synonymous mutations were in genes responsible for flagella (*n*=7) and iron transport (*n*=2) (Table S4), although no motility changes were detected in this isolate. Eleven SNVs separated 201000305 and 09–9322 (8 non-synonymous SNVs, 2 synonymous SNVs, 1 intergenic SNV). One of these eleven SNVs was found in another iron transport gene (asmbl_3909, Table S4).

### SGI in *S*. *e* *nterica* serotype Kentucky

**Fig. 2.**
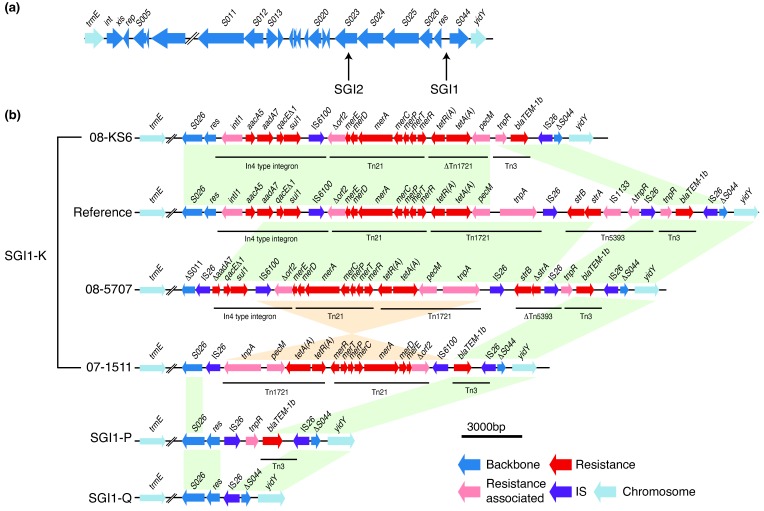
SGI variation in *S.* *e* *nterica* serotype Kentucky ST198. (a) Backbone of SGI, with arrows pointing to the different insertion sites of the resistance region in SGI1 and SGI2. (b) Different examples of SGI1 types in *S*. *e* *nterica* serotype Kentucky ST198. Arrows show ORFs of the SGI backbone and MDR region with arrowheads indicating direction of transcription; colour indicates gene class. Coloured blocks indicate regions of homology between sequences in the same orientation: green, same orientation; orange, inverse orientation.

The presence of any SGI backbone genes was taken as evidence of SGI integration (Fig. S3). The data indicate that the SGI has been acquired by *S*. *e* *nterica* serotype Kentucky ST198 on three distinct occasions, integrating each time site-specifically in the 3′-end of the *trmE* gene. SGI2 (previously SGI1-J), which carries the multidrug-resistance region in a different position of the SGI1 backbone (Fig. 2a), was present in a single isolate from Indonesia, and SGI1-B was present in a single isolate from India; both these isolates were distantly related to the main MDR lineage (Fig. 1). The vast majority (95 %) of genomes belonging to the main MDR lineage carried the SGI1-K subtype or one of its derivatives (SGI1-P or SGI1-Q), consistent with acquisition of SGI1-K in the MRCA circa 1989 in Egypt, shortly before the expansion of the clone (Fig. 1). Within this MDR lineage, some isolates had large deletions of the SGI backbone (e.g. deletions spanning from *S011* to *S026*, or from *int* to *S026*), but still retained the multidrug-resistance region between *trmE* and *yidY* (Figs S3 and S4).

Almost every SGI1-positive *S*. *e* *nterica* serotype Kentucky ST198 isolate in this study had a distinct SGI structure (Figs 2b and S4). In addition to large deletions of the SGI backbone, some isolates had inversions of whole or part of the resistance gene segment of the island, with various deletions and rearrangements of the transposons (Fig. 2b). There were multiple different IS*26* insertion sites within the resistance elements of the island, providing evidence that IS*26* has mediated the majority of differences found in the resistance region of the island (Fig. 2b). We found that IS*26* was rarely present in *S*. *e* *nterica* serotype Kentucky ST198 isolates outside of the MDR lineage (Fig. S3). Within the MDR lineage, linear regression analysis of IS*26* copy number against year of isolation showed some evidence of IS*26* accumulation over time (0.12 IS*26* copies per year, *P*=0.01, *R* ^2^=0.05) (Fig. S5).

There was no relationship between degraded SGI1s and geographical region or country, or between the loss of core SGI resistance genes (defined as *aacA5, bla*_TEM-1_ *, sul1* and *tetA*) and region (see Methods). We found that *strAB*, *aphA2, aph3-Ia*, *catA1, dfrA12* and *mph(A*) were present significantly more frequently in Egypt compared to all other regions (Table S5).

### Multidrug-resistance genes and plasmids in *S*. *e* *nterica* serotype Kentucky ST198

**Fig. 3.**
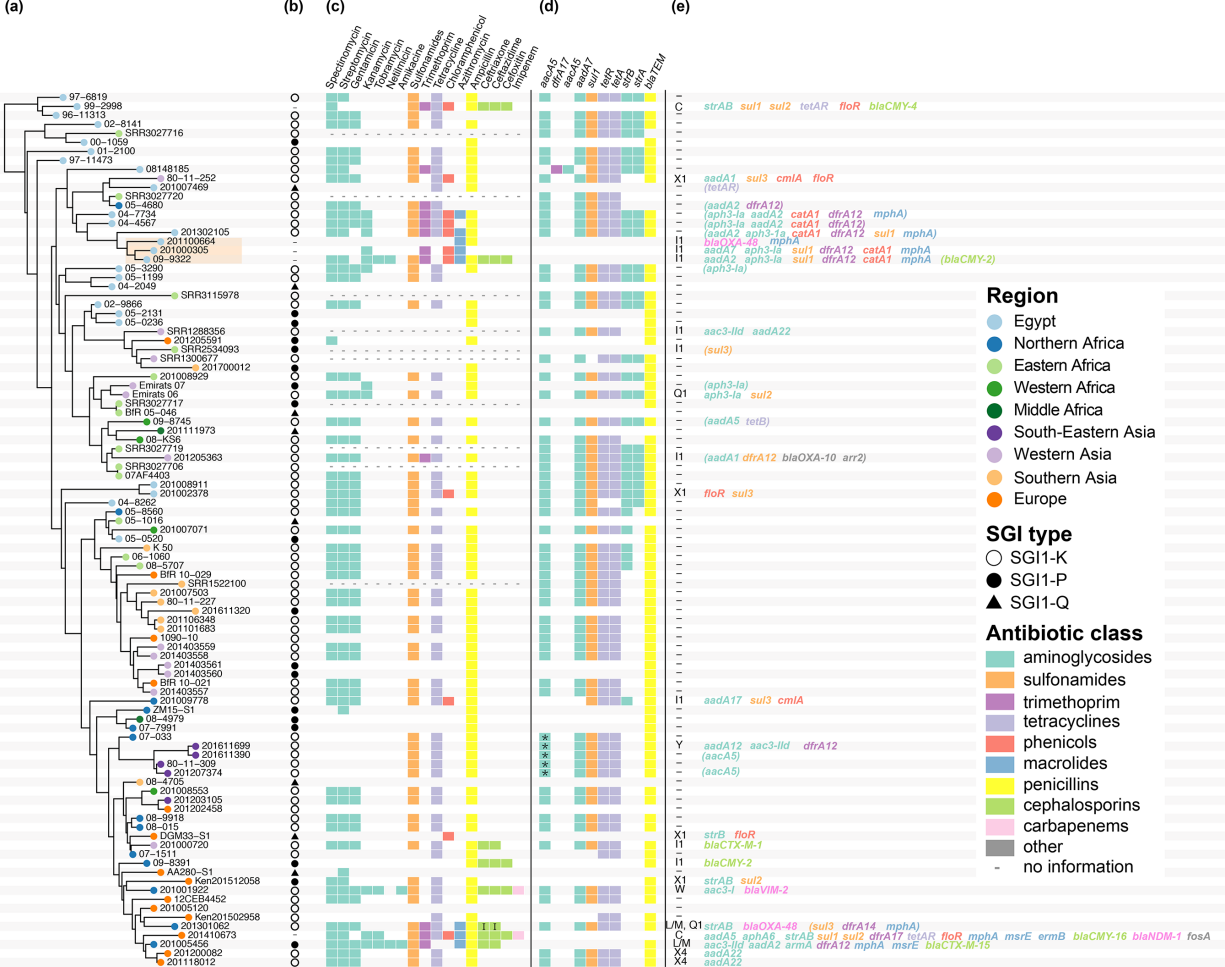
Horizontally acquired AMR genes in the *S.* *e* *nterica* serotype Kentucky ST198 MDR lineage. (a) Dated Bayesian (beast) phylogeny for the MDR lineage, extracted from the tree shown in Fig. 1. Leaf nodes are coloured by region of origin (see the key); the orange box highlights three isolates recovered from the same patient over 3 years. (b–e) AMR features of each isolate in the tree. (b) SGI type (see the key, dash indicates no SGI detected). (c) AMR phenotypes, indicated as boxes coloured by antimicrobial class (see the key, I in the box denotes intermediate resistance). (d) AMR genes located within the SGI1 are indicated with boxes coloured by antimicrobial class (* in the box indicates gene is interrupted). (e) Plasmid incompatibility group(s) identified in each genome; AMR genes located within these plasmids are printed, coloured by antimicrobial class; genes in brackets are genes for which it was not possible to determine location.

Overall, we found that 35 isolates in the full strain set carried at least one plasmid, covering 13 different known plasmid incompatibility types (Table S1). Within the MDR lineage, there was extensive phenotypic and genotypic variation in antimicrobial susceptibility observed (Fig. 3). A part of this variability could be attributed to the acquisition of plasmids carrying additional AMR genes, as 32 isolates in the MDR lineage carried genes outside the SGI that are likely plasmid-borne (Fig. 3e). Known plasmid replicons were identified in 23 isolates, and in total we identified eight different plasmid incompatibility types across the MDR strain set (C, I1, L/M, Q1, W, X1, X4, Y). From these 23 isolates carrying known plasmid incompatibility types, we were able to determine precise plasmid–AMR gene links for 20 isolates.

There appeared to be no link between geography and plasmid type, with plasmids present in isolates from multiple different regions (Fig. S6). The majority of genes encoding carbapenemases (*bla*_OXA-48_ and *bla*_NDM-1_), ESBLs (*bla*_CTX-M-1_) and cephamycinases (*bla*_CMY-2_, *bla*_CMY-4_ and *bla*_CMY-16_) were carried by either IncI1 or IncC (previously IncA/C_2_) plasmids (Fig. 3d, e). Two IncL/M plasmids were found to carry *bla*_OXA-48_ or *bla*_CTX-M-15_ and an IncW plasmid was found to carry *bla*_VIM-2_ (Fig. 3d, e). The eight isolates resistant to azithromycin contained the *mph(A*) gene. These isolates clustered into two groups. A plasmid location of *mph(A*) was found for four isolates. Three different Inc types were identified (IncI1, IncC and IncL/M).

There was little evidence that any plasmids were being maintained as the MDR lineage evolved (Fig. 3), although the group of three isolates recovered from the same patient in Egypt (09–9322, 201000305, 201100664; discussed above) all carried IncI1 plasmids. These three plasmids were identical in their core gene content, although IncI1 plasmids in 201100664 differed from those in the earlier two isolates by two intergenic SNVs (Fig. S7). Interestingly, these three isolates all lacked the SGI and any other chromosomal resistance genes, and their IncI1 plasmids differed substantially from one another in resistance gene content (Fig. 3e). The two early isolates mostly carried resistance genes for aminoglycosides, sulfonamides, trimethoprim, phenicols and macrolides. The plasmid in the final isolate, 201100664, had lost almost all of the resistance genes found in the previous two isolates, except for *mph(A*), and had gained the carbapenemase-encoding *bla*_OXA-48_ gene. IncI1 plasmids were detected in a further six *S*. *e* *nterica* serotype Kentucky ST198 genomes, but these did not cluster in either the IncI1 plasmid tree or the chromosome tree, consistent with seven distinct introductions of IncI1 plasmids into the *S*. *e* *nterica* serotype Kentucky ST198 MDR lineage, each associated with distinct AMR gene contents (Figs 3 and S7).

Two isolates of the MDR lineage carried IncC plasmids (99–2998 and 201410673). Both IncC plasmids were genotyped as pST3, which is commonly associated with *bla*_CMY_ [58], and this cephamycinase-encoding gene was found in the plasmid from isolate 99–2998. Interestingly, the IncC plasmid in isolate 201401673 was carrying a carbapenemase-encoding *bla*_NDM-1_ gene, which is more commonly found in pST1 IncC plasmids [58]. This *bla*_NDM-1_ gene was found in a different structural context to the *bla*_NDM_ genes in the pST1 IncC plasmids; as usual it was downstream of IS*Aba125*; however, instead of being upstream of *ble*, it was upstream of *qacEΔ1* and *sul1*, with a remnant of the *ble* gene left behind from the insertion of *qacEΔ1* (Fig. S8). We found that this *bla*_NDM-1_ region was entirely covered by WGS reads, with no breaks or gaps in coverage, supporting that it is the true structure in this plasmid (Fig. S8). This configuration also appears in another pST3 IncC plasmid, pRH-1238, from *S. enterica* serotype Corvallis (GenBank accession number KR091911), isolated from a wild bird in Germany [65].

Another source for the phenotypic diversity of *S*. *e* *nterica* serotype Kentucky ST198 susceptibility profiles was variations in the SGI1 (Fig. 3d). Notably, plasmid carriage was significantly associated in the cases where SGI1-P, SGI1-Q (containing few or no AMR genes) or no SGI were detected (Fisher’s exact test, *P*=0.024, odds ratio=2.65, 95 % confidence interval=1.09–6.64) (Fig. 3b, d and e).

### Chromosomal gene content diversity amongst *S*. *e* *nterica* serotype Kentucky ST198 isolates

There was very little gene content diversity evident amongst the *S*. *e* *nterica* serotype Kentucky ST198 chromosome sequences (Fig. S9). Three phages were detected within the reference genome 201001922 and these three phage regions, in addition to the SGI1, were the only regions to show large differences between genomes from the MDR lineage and those from other lineages (Fig. S9). Supporting this, within the accessory gene content identified using Roary (see Methods), only four genes were found to be present exclusively in all but one of the MDR lineage genomes. All four of these genes were located within a single phage, ST160 (43 kbp, 46 genes, positions 541864–584944 in the 201001922 reference genome). This phage was found to be inserted between *ompP* and *mlaA* in the MDR lineage. A variation of this phage was also present in the oldest genome, 98K, which is outside the MDR lineage; however, in this genome the phage was inserted between *napB* and *hutI*.

Examination of the virulence gene content in all isolates revealed that there was no difference between *S*. *e* *nterica* serotype Kentucky ST198 isolates belonging to the MDR lineage and those belonging to other lineages (Fig. S10). Only five virulence genes were present in less than 95 % of genomes – *gogB* (0.8 %), *sipB* (7 %), *sipC* (35 %), *ompD* (57 %) and *sciQ* (80 %) (Table S6) – however, these were randomly distributed in the tree and not associated with lineage (Fig. S10).

## Discussion

Our data show that nearly all MDR *S*. *e* *nterica* serotype Kentucky ST198 belong to a single lineage that has accumulated AMR determinants since the early 1990s (Fig. 1). It first acquired a variant of the SGI1, SGI1-K, which conferred resistance to ampicillin, streptomycin, gentamicin, sulfamethoxazole and tetracycline (Fig. 2). The SGI1 structure appears to be highly susceptible to genetic rearrangements, with distinct forms found in each isolate likely due to the transpositional activity of IS*26*, which resulted in deletion of some or all genes inside SGI1. The loss of resistance genes was often made up for by acquisition of additional MDR plasmids (Fig. 3).

IS*26* is 820 bp long and encodes a single transposase with 14 bp terminal repeats on each end [66]. Each of the three SGI1 subtypes found in the MDR lineage carried one or more copies of IS*26*, and all genomes in the MDR lineage carried IS*26*, with no genomes outside of this clade carrying IS*26*. The recently described mechanism used by IS*26* to transpose may provide an explanation as to why the SGI variants in these isolates are so dynamic. During the transposition, IS*26* extracts itself from the donor DNA molecule, as well as DNA lying upstream of it between itself and another IS*26* element, and uses this to form a translocatable unit [67]. It then finds another IS*26* element in the receiving DNA molecule, and inserts itself as well as the excised donor DNA next to it, forming a tandem array of IS*26*s in direct orientation [67]. This model illustrates that IS*26* is likely the causative agent for many of the deletions, inversions and transpositions within the SGI, eventually resulting in the genesis of the different SGI1 variants (SGI1-K, SGI1-P and SGI1-Q) seen in this dataset (Fig. 2).

Whilst the origin of the MDR clade appears to be intimately linked with the acquisition of the SGI1 in Egypt, it is the QRDR triple-mutant CIP^R^ subclade that disseminated globally (Fig. 1). Ciprofloxacin resistance is infrequent in *Salmonella* [68], and we hypothesize that this high-level resistance is linked to strong selective pressure exerted by fluoroquinolone use in poultry, *S*. *e* *nterica* serotype Kentucky’s main reservoir [69]. This resistance might also have come at no cost to the fitness of the bacterial cell, as has been shown in close relatives *S*. *e* *nterica* serotype Typhi and *Escherichia coli* [70, 71].

During its spread around the world, the *S*. *e* *nterica* serotype Kentucky ST198 MDR lineage became more resistant by the additional acquisition of various AMR plasmids, carrying genes encoding resistance to newer drugs, including third-generation cephalosporins, carbapenems and azithromycin. These genes were acquired locally around the Mediterranean basin with no subsequent clonal expansion. Interestingly, the two isolates containing IncC plasmids did not carry the SGI. This observation is supported by many studies in the literature that have described the incompatibility of the SGI and IncC plasmids, as they share the same regulatory system [14, 72, 73].

In this study, we were unable to detect any other non-AMR related genes that could explain the clonal success of the MDR lineage. Examination of phage, pseudogenes and known virulence genes did not reveal any significant differences between the MDR lineage and other *S*. *e* *nterica* serotype Kentucky ST198 genomes, although this does not rule out the possibility of more subtle variants contributing to virulence such as the regulatory SNV recently described for invasive *S*. *e* *nterica* serotype Typhimurium ST313 [74].

In conclusion, WGS analysis of *S*. *e* *nterica* serotype Kentucky ST198 has significantly expanded our knowledge of the evolution and dissemination of MDR variants of this important pathogen. Previously, as this lineage was emerging, MLST and PFGE were used in combination [2, 3] for this purpose; however, the diversity of PFGE types of CIP^R^ *S*. *e* *nterica* serotype Kentucky ST198 isolates precluded any fine-scale or long-term analysis of *S*. *e* *nterica* serotype Kentucky ST198 dissemination, which our data shows was likely due to noise introduced by IS*26* activity. The population structure uncovered here should serve as a useful framework with which to understand and track the ongoing evolution of the MDR lineage of *S*. *e* *nterica* serotype Kentucky ST198, which our data clarifies is a globally disseminated clone capable of rapid spread and further accumulation of last-line AMR determinants.

## Data Bibliography

1. Hawkey J., ENA, PRJNA445436 (2018).
  2. Hawkey J., GenBank, CP028357 (2018).

## Supplementary Data

Supplementary File 1

Supplementary File 2
