# Supplementary material for: Global phylogenomics of multidrug-resistant Salmonella enterica serotype Kentucky ST198
Source: Microb Genom. 2019 May 20;5(7):e000269. doi: 10.1099/mgen.0.000269 (PMC6700661; doi:10.1099/mgen.0.000269)
Supplement: Supplementary File 1 [file mgen-5-269-s001.pdf]

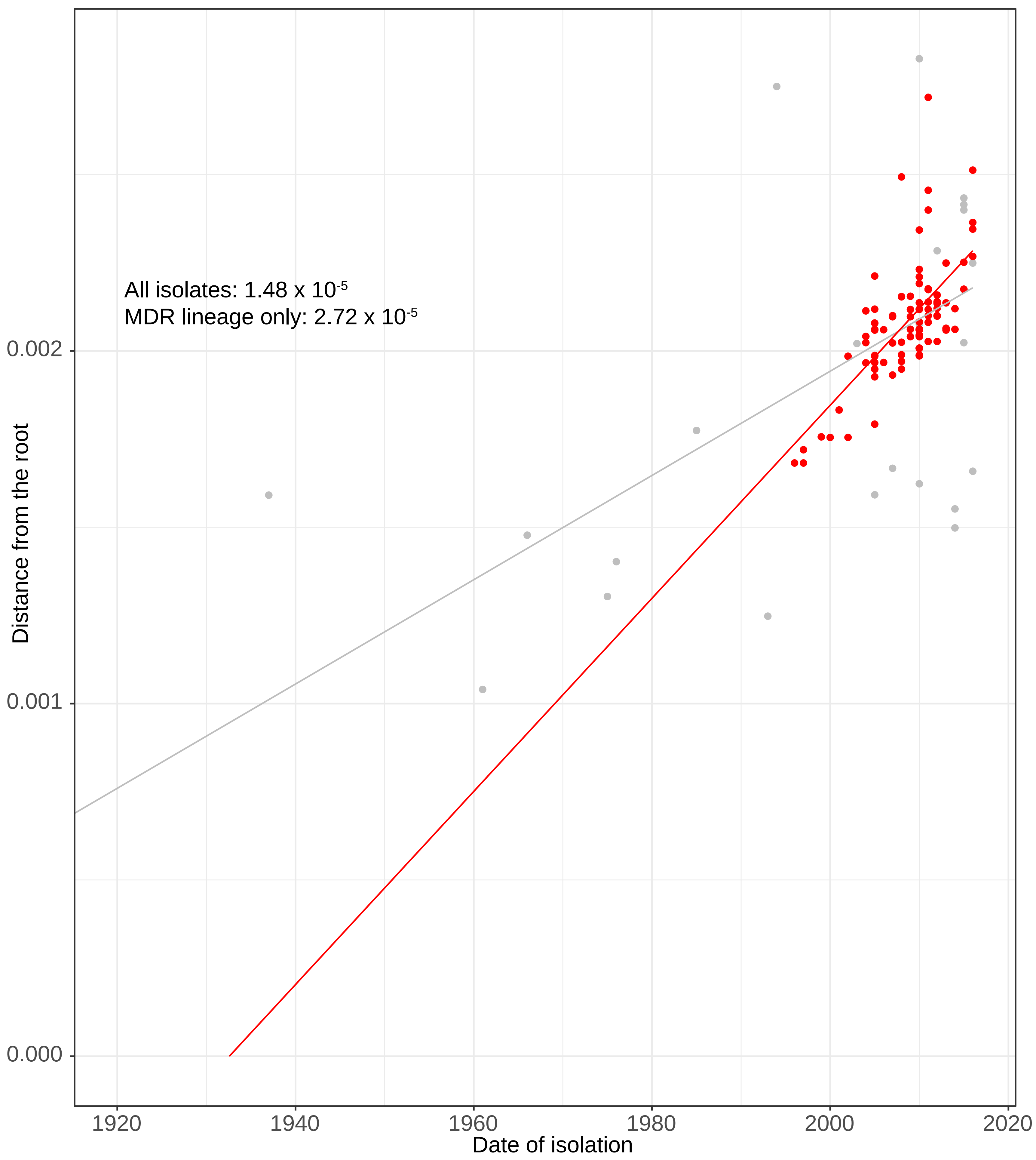

Supplementary Figure 1: Temporal signal in the maximum likelihood phylogeny.
Grey dots, isolates not within the MDR lineage; red dots, isolates within the MDR lineage.
Grey line, linear regression of all isolates in tree; red line, linear regression of only isolates within the MDR lineage. Values reported are the correlation coefficients for these regressions.

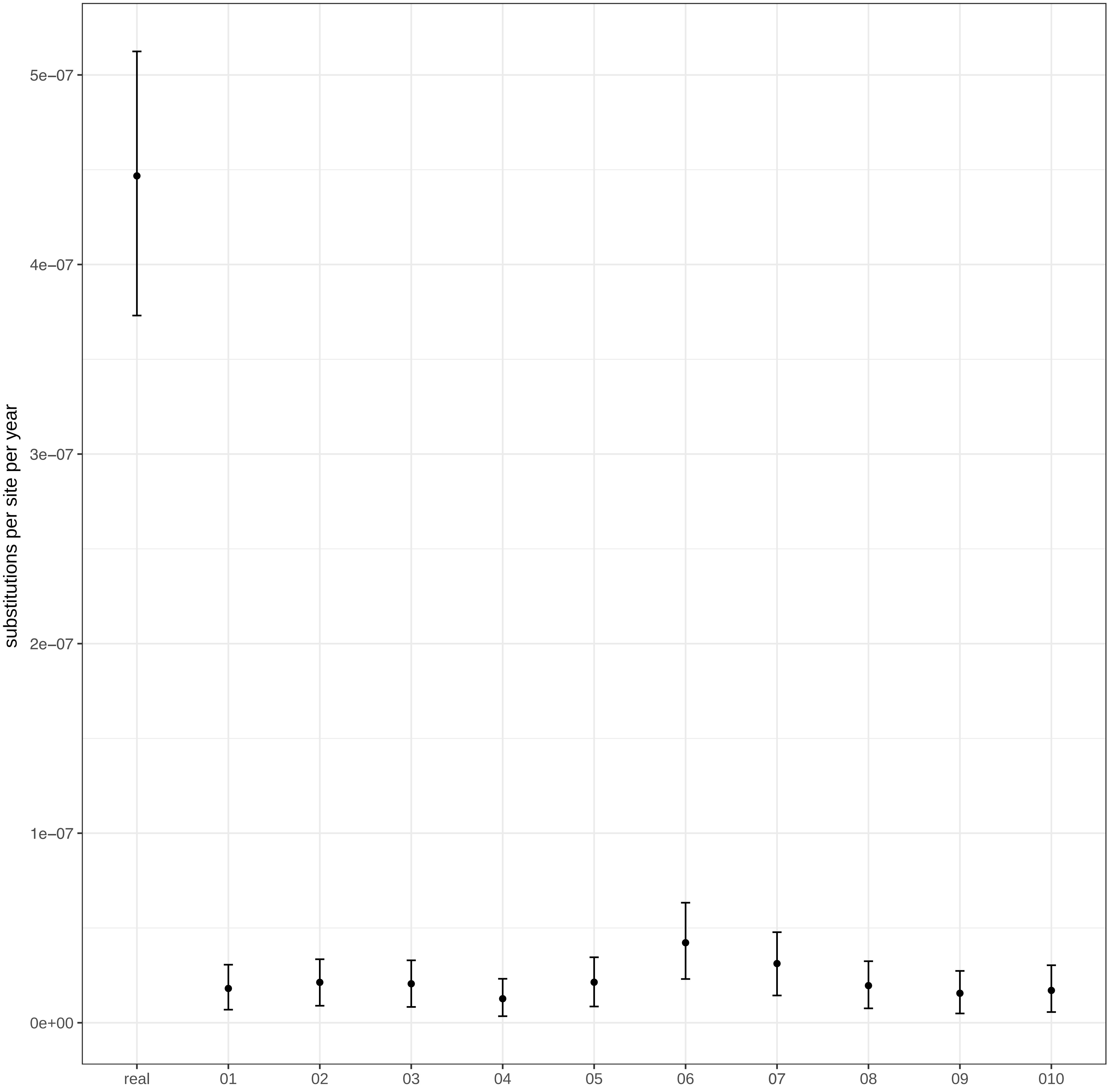

Supplementary Figure 2: Mutation rate estimates for real and randomised tip dates in S. Kentucky ST198. First column, real mutation rate, in substitutions per site per year. Subsequent columns show mutation rate when tip dates are randomised. Black circles are the mean rate estimated by BEAST, with error bars showing 95% highest posterior density (HPD).

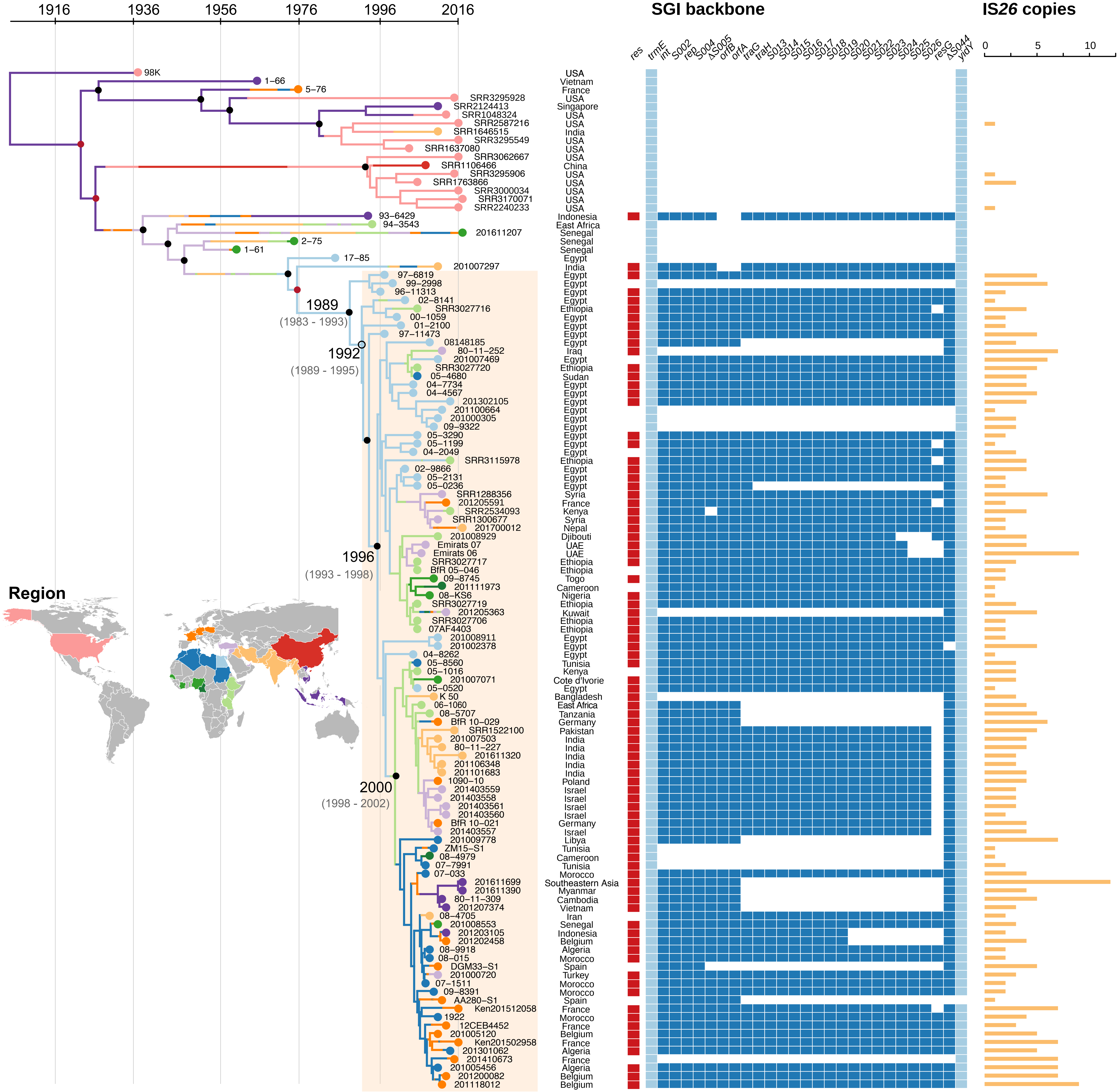

Supplementary Figure 3: Presence of SGI backbone genes in each S. Kentucky ST198 isolate, with an estimate of overall IS26 copy number. Left, dated Bayesian phylogeny, with tips and branches coloured by region (as per map inset and Figure 1). Red blocks indicate the presence at least one resistance gene in the SGI resistance region. Light blue blocks indicate presence of chromosomal genes flanking the SGI (trmE and yidY). Dark blue blocks indicate presence of SGI backbone genes. Yellow bars show estimated IS26 copy number in each isolate.

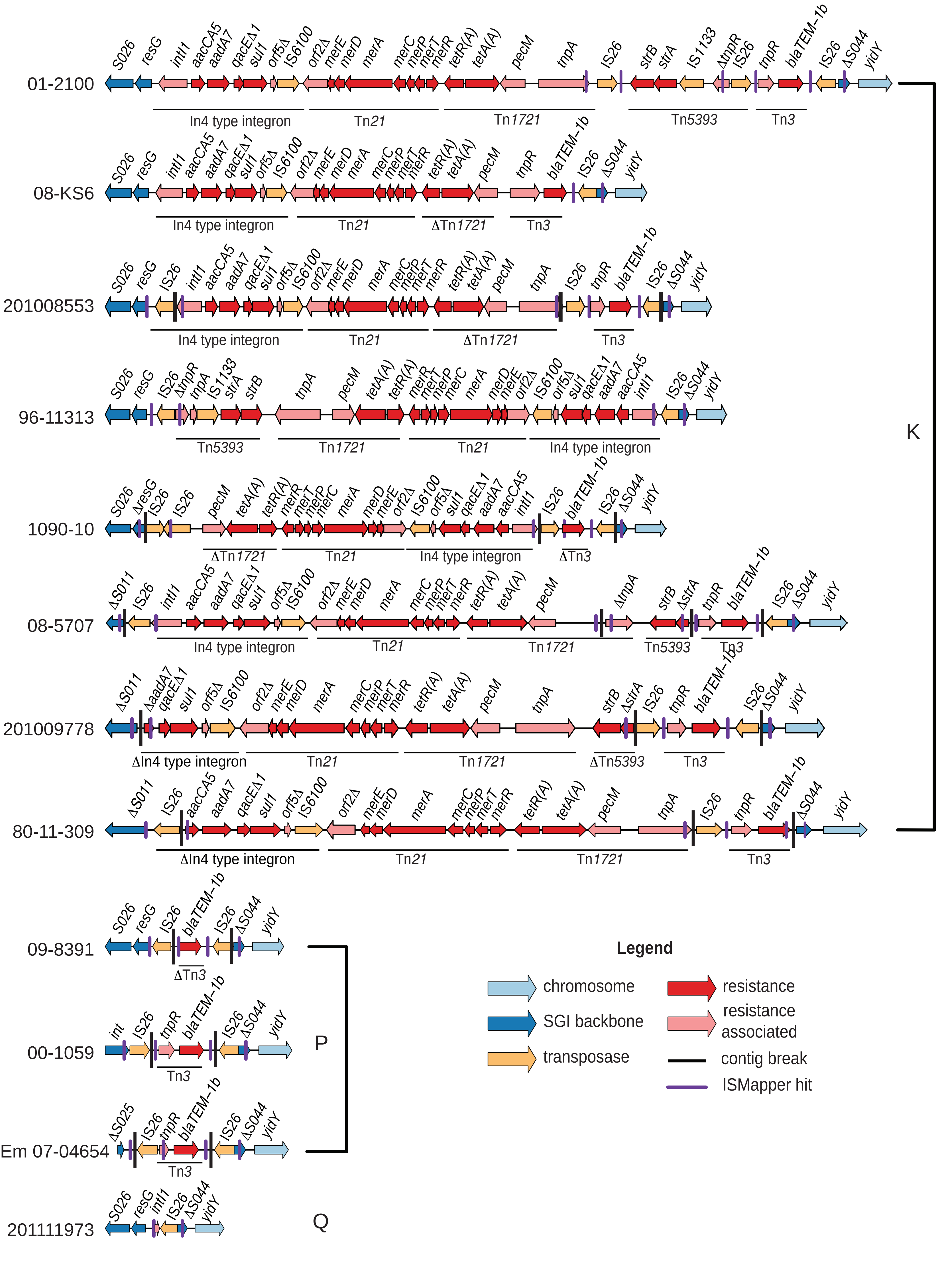

Supplementary Figure 4: Additional SGI1 examples.
Coding regions are represented as arrows and are coloured as per legend. SGI sequences are grouped by type - SGI1-K, SGI1-P and SGI1-Q. Contig breaks are shown by thick black vertical lines, with IS26 flanking regions detected with ISMapper marked with purple vertical lines.

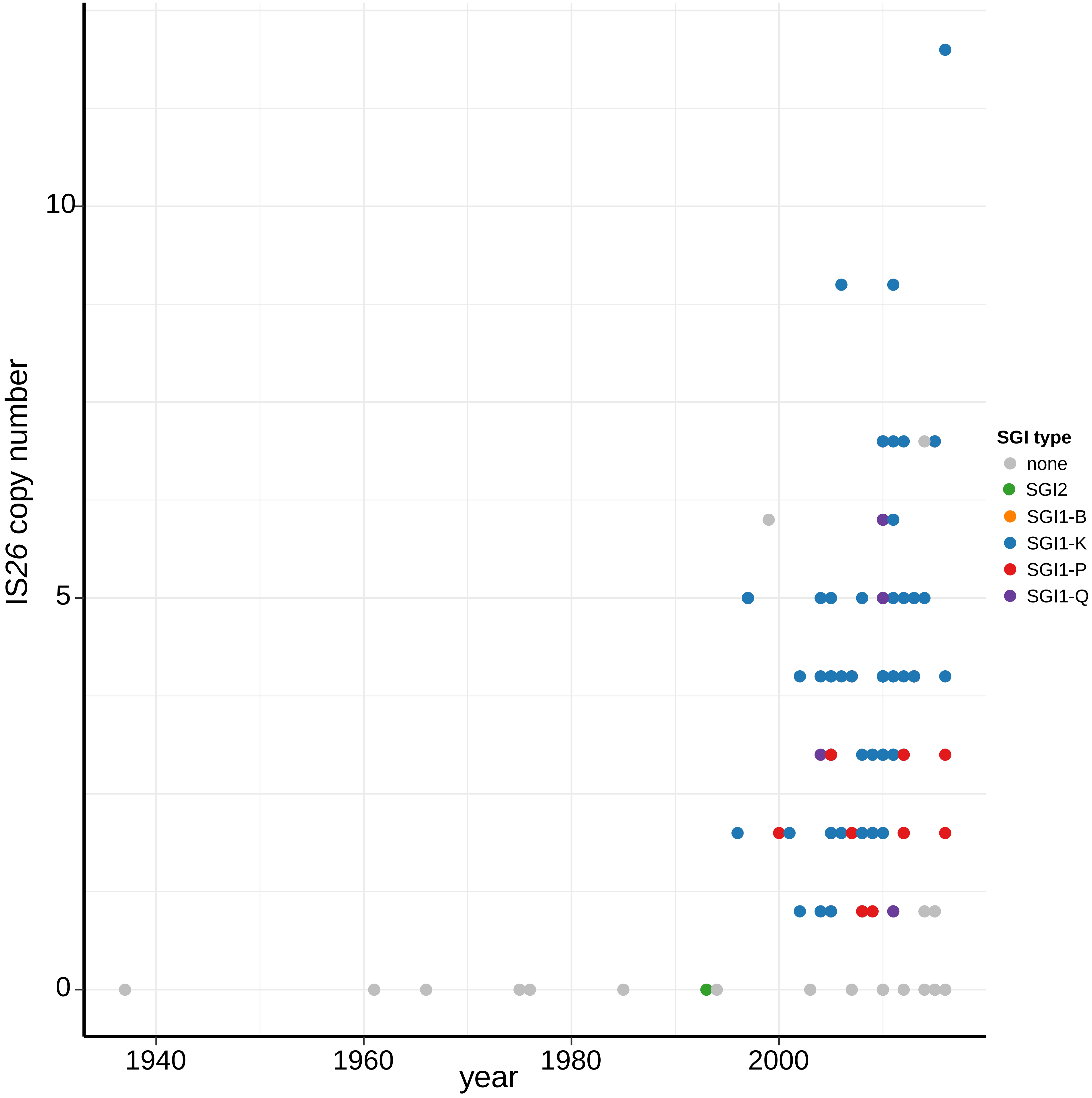

Supplementary Figure 5: Relationship between year of isolation of S. Kentucky ST198 and estimated IS26 copy number.
Points are coloured by the SGI type found in that strain, as per legend.

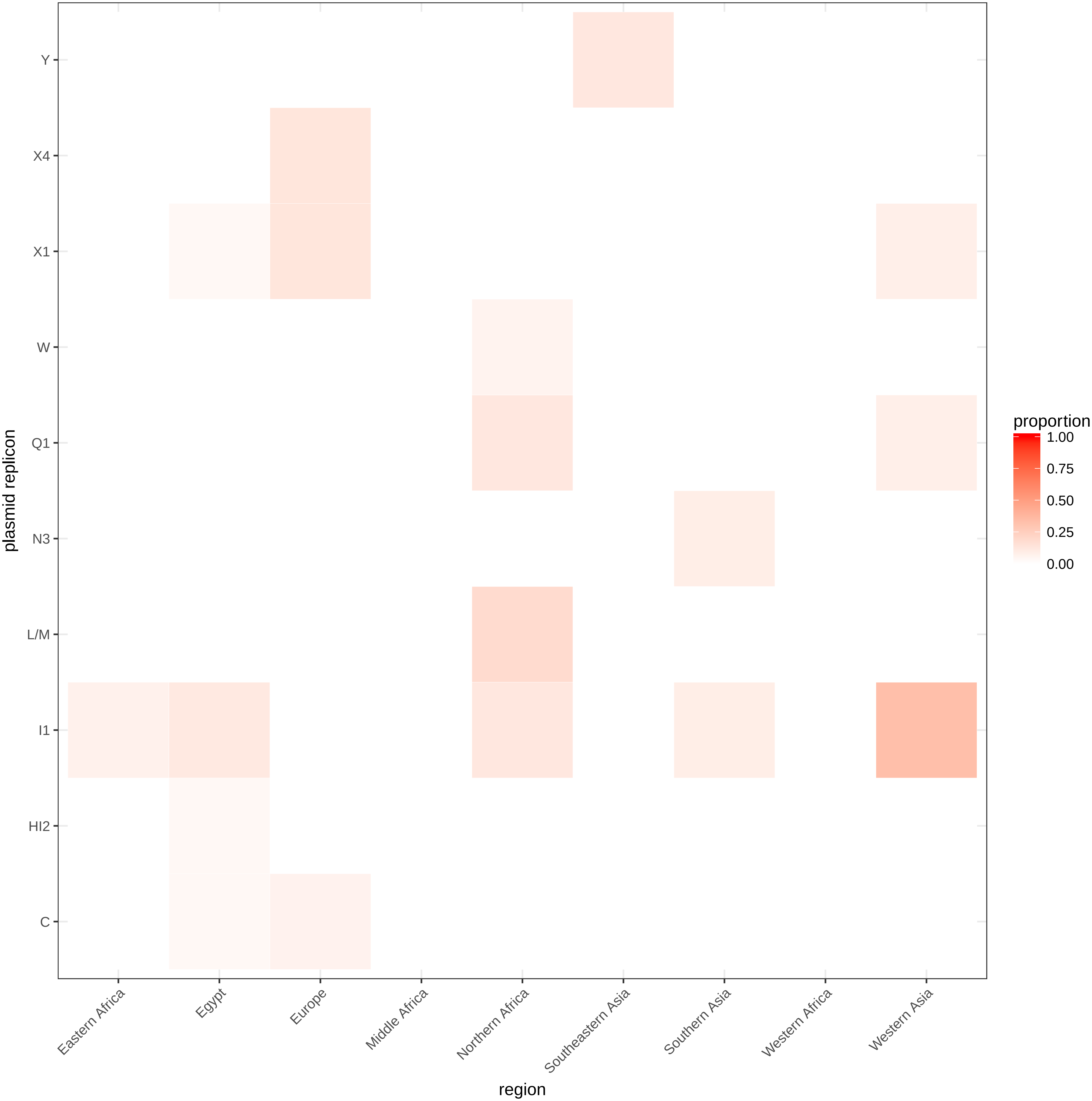

Supplementary Figure 6: Heatmap showing proportion of each plasmid replicon within each region found in the S. Kentucky ST198 MDR lineage.

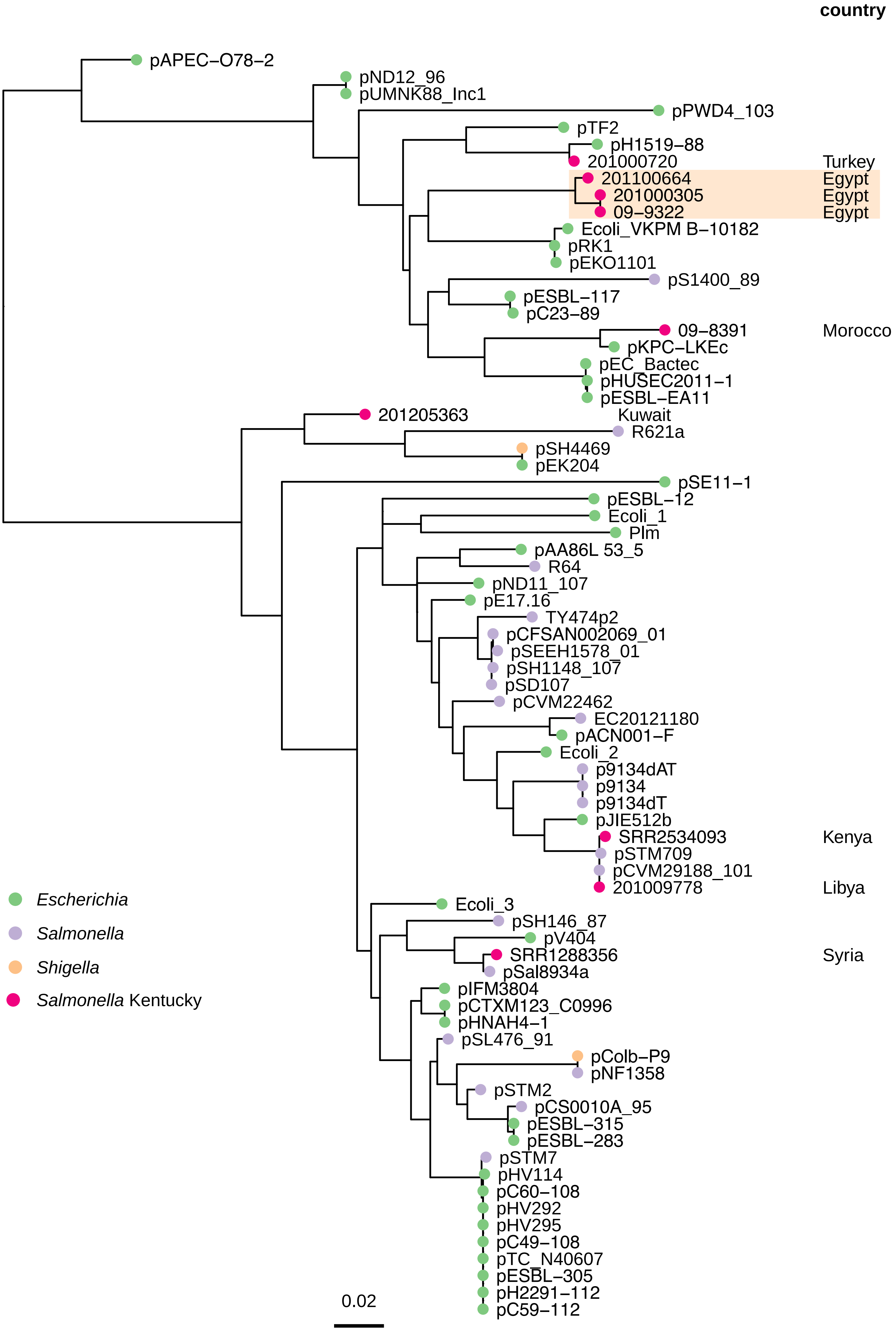

Supplementary Figure 7: Maximum-likelihood phylogeny of IncI1 plasmids. Phylogeny is midpoint rooted, with tips coloured by genus (as per legend) and S. Kentucky ST198 isolates from this study coloured pink. Orange box indicates three S. Kentucky isolates from a single patient, all carrying IncI1 plasmids. Country of isolation is listed next to the S. Kentucky isolates used in this study.

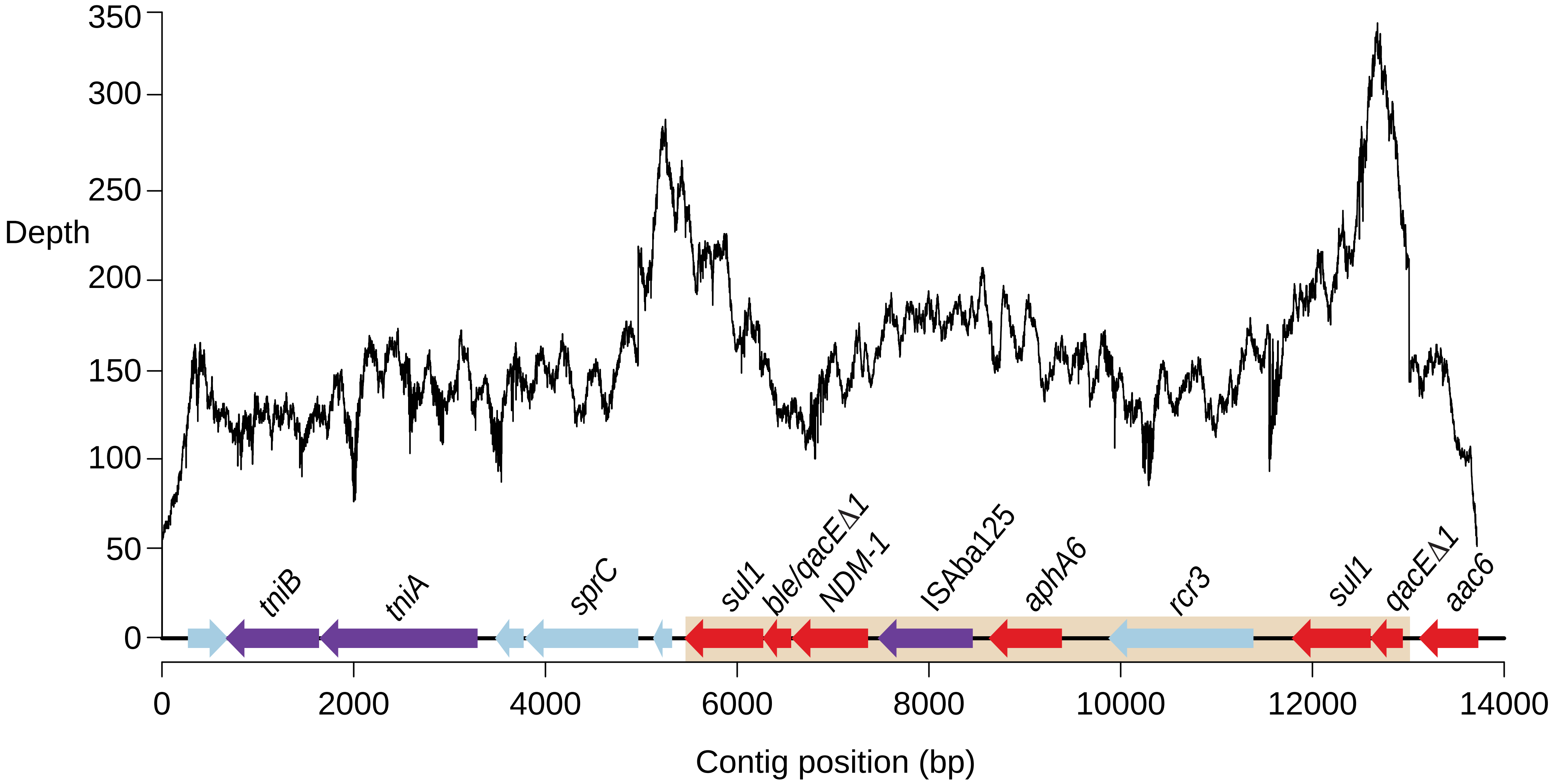

Supplementary Figure 8: Coverage across NDM-1 region in isolate 201410673.
Positions of genes depicted as arrows, with arrows pointing in the direction of transcription.
Light blue, other plasmid genes; red, resistance genes; purple, transposable elements. Genes without names are hypothetical proteins. Dark orange box shows boundaries of exact match to the same blaNDM-1 configuration in S. enterica serotype Corvallis (GenBank accession no. KR091911).
Depth is number of reads at each base position on the x-axis.

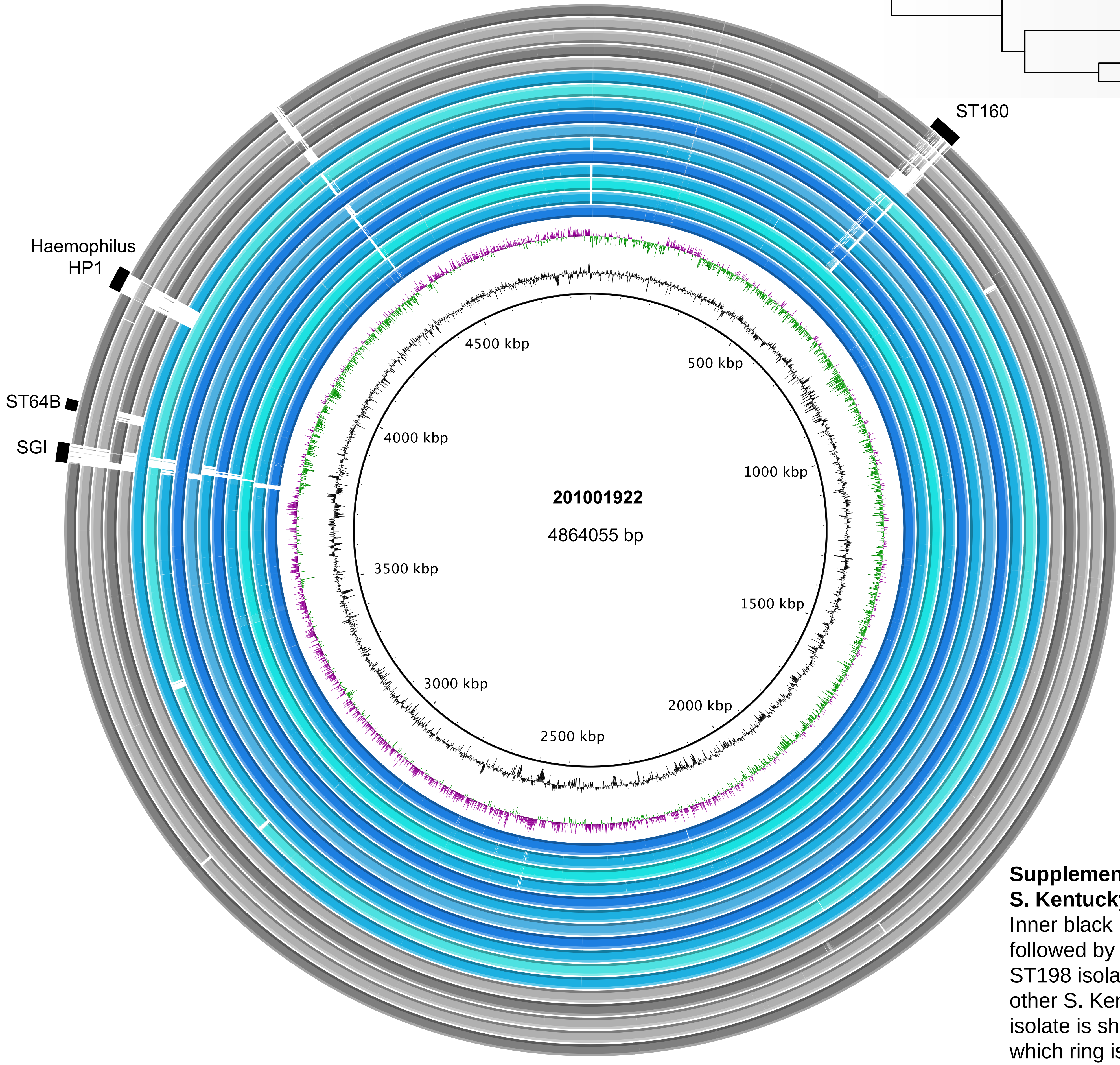

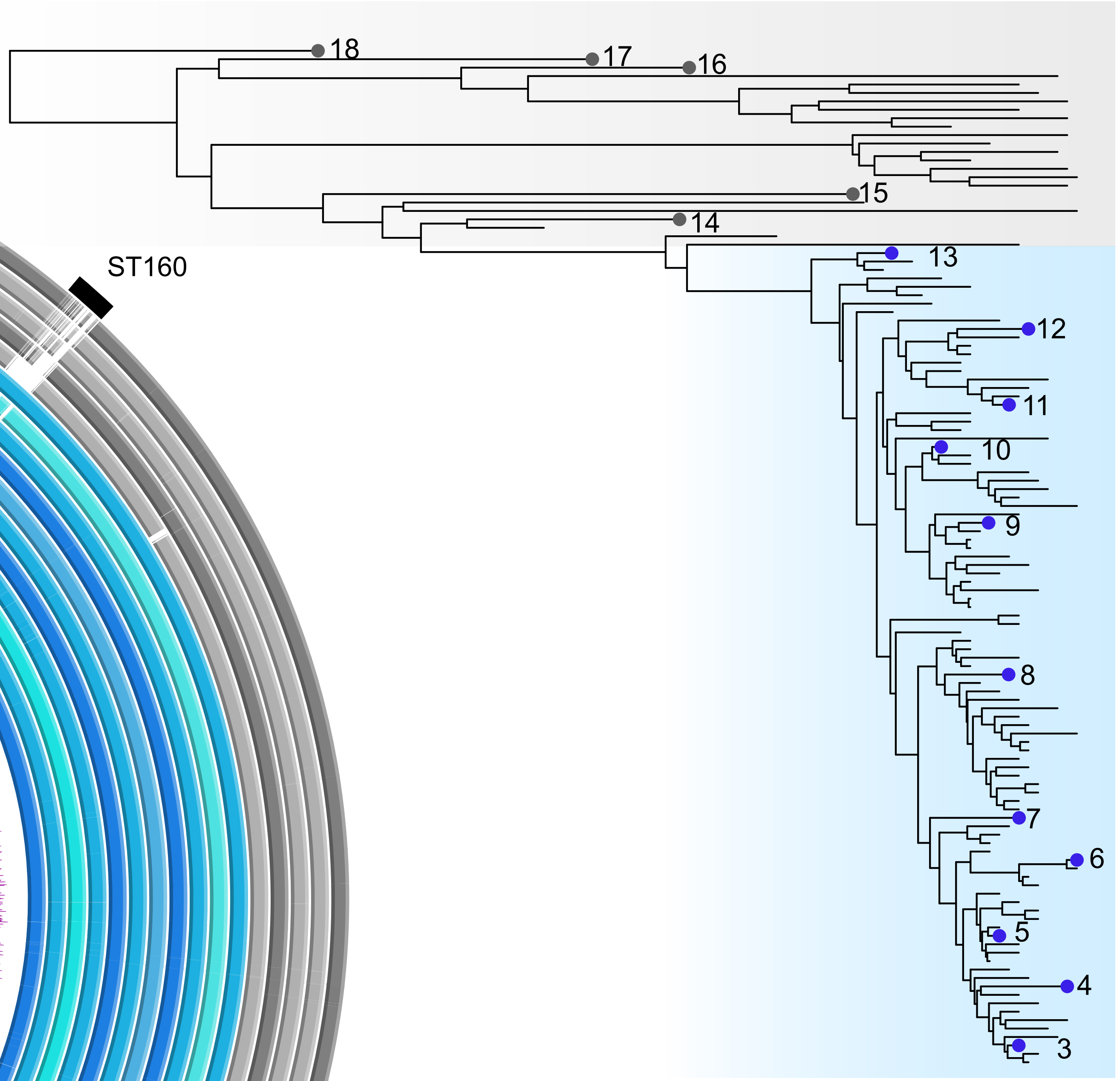

Supplementary Figure 9: Comparison of a subset of S. Kentucky ST198 genomes to reference genome 201001922. Inner black ring indicates 201001922 genome position, followed by GC content. Blue rings indicate S. Kentucky ST198 isolates belonging to the MDR lineage, grey rings other S. Kentucky ST198 isolates. The location of each isolate is shown on the phylogeny - numbers indicate which ring is shown by the isolate.

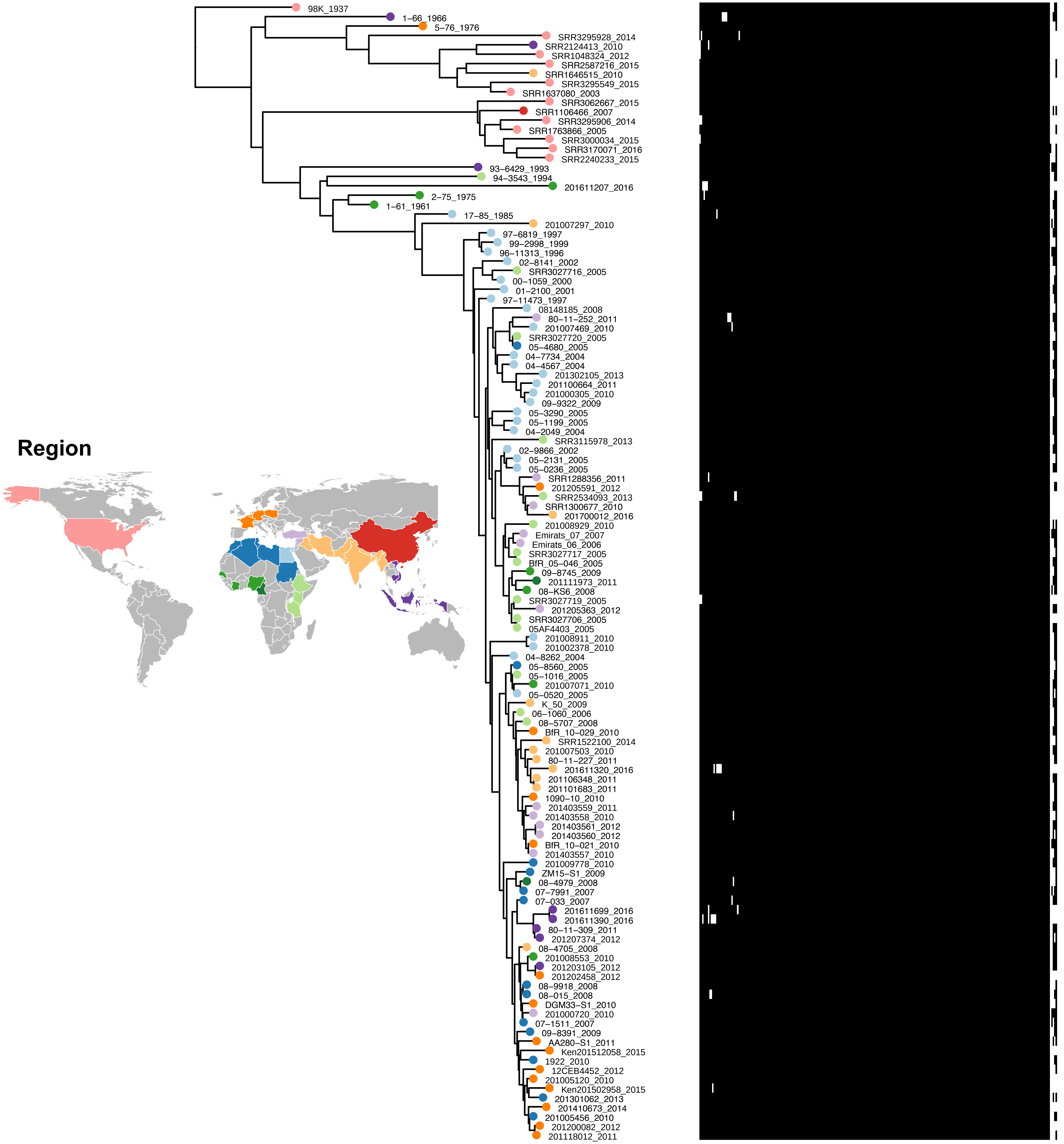

Supplementary Figure 10: Heatmap of virulence gene content in all S. Kentucky ST198 isolates. Tips of the phylogeny are coloured by region, as per legend. Each column of the heatmap is a virulence gene, black indicates presence, white indicates absence. Virulence genes are clustered to reveal patterns.
